# Potassium bis­(1,1,1,5,5,5-hexa­fluoro­pentane-2,4-dionato)bis­(4,4,4-trifluoro-1-phenyl­butane-1,3-dionato)europate(III)

**DOI:** 10.1107/S1600536810021458

**Published:** 2010-06-16

**Authors:** Julie M. Stanley, Bradley J. Holliday

**Affiliations:** a Department of Chemistry and Biochemistry, The University of Texas at Austin, 1 University Station, A5300, Austin, Texas 78712, USA

## Abstract

In the crystal structure of the title complex, K[Eu(C_5_HF_6_O_2_)_2_(C_10_H_6_F_3_O_2_)_2_], the Eu^III^ ion is in a slightly distorted square-anti­prismatic coordination geometry which is defined by eight O atoms of the anionic β-diketone ligands. The two K^+^ ions lie on crystallographic inversion centers. The Eu—O bond distances are in the range 2.294 (5)–2.413 (5) Å. The crystal used was a non-merohedral twin, the ratio of the twin domains being 0.5236 (5):0.4764 (5).

## Related literature

For general background to and potential applications of luminescent lanthanide complexes containing β-diketonates, see: Eliseeva & Bunzli (2010 ▶); de Bettencourt-Dias (1997 ▶); Stanley *et al.* (2010 ▶); Chen & Holliday (2008 ▶). For similar structures, see: Nockemann *et al.* (2005 ▶); Burns & Danford (1969 ▶). The twin law was determined using *TwinSolve* (Rigaku/MSC, 2002 ▶).
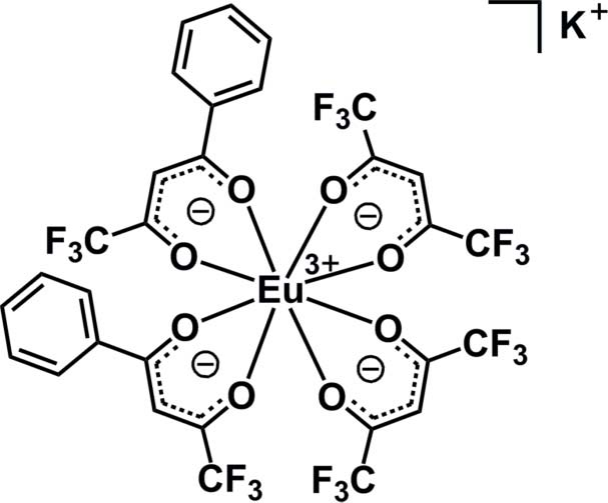


## Experimental

### 

#### Crystal data

K[Eu(C_5_HF_6_O_2_)_2_(C_10_H_6_F_3_O_2_)_2_]
*M* *_r_* = 1035.47
Triclinic, 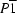

*a* = 11.737 (2) Å
*b* = 12.468 (2) Å
*c* = 13.788 (3) Å
α = 68.457 (8)°
β = 71.791 (8)°
γ = 71.726 (8)°
*V* = 1737.1 (5) Å^3^
*Z* = 2
Mo *K*α radiation
μ = 2.07 mm^−1^
*T* = 100 K
0.12 × 0.06 × 0.03 mm

#### Data collection

Rigaku AFC12 with Saturn 724+ CCD diffractometer
Absorption correction: multi-scan (*ABSCOR*; Higashi, 2001 ▶) *T*_min_ = 0.789, *T*_max_ = 0.941
25336 measured reflections
25336 independent reflections
22905 reflections with *I* > 2σ(*I*)

#### Refinement

*R*[*F* ^2^ > 2σ(*F* ^2^)] = 0.096
*wR*(*F* ^2^) = 0.197
*S* = 3.00
25336 reflections
527 parameters
H-atom parameters constrained
Δρ_max_ = 3.65 e Å^−3^
Δρ_min_ = −3.79 e Å^−3^

### 

Data collection: *CrystalClear* (Rigaku/MSC, 2008 ▶); cell refinement: *CrystalClear*; data reduction: *CrystalClear*; program(s) used to solve structure: *SIR97* (Altomare *et al.*, 1999 ▶) within *WinGX* (Farrugia, 1999 ▶); program(s) used to refine structure: *SHELXL97* (Sheldrick, 2008 ▶); molecular graphics: *ORTEP-3* (Farrugia, 1997 ▶) and *POV-RAY* (Persistence of Vision, 2004 ▶); software used to prepare material for publication: *SHELXL97*.

## Supplementary Material

Crystal structure: contains datablocks I, global. DOI: 10.1107/S1600536810021458/lh5047sup1.cif

Structure factors: contains datablocks I. DOI: 10.1107/S1600536810021458/lh5047Isup2.hkl

Additional supplementary materials:  crystallographic information; 3D view; checkCIF report

## Figures and Tables

**Table 1:** Selected bond lengths (Å)

Eu1—O4	2.294 (5)
Eu1—O6	2.315 (5)
Eu1—O5	2.367 (4)
Eu1—O3	2.368 (4)
Eu1—O8	2.394 (5)
Eu1—O2	2.400 (5)
Eu1—O7	2.412 (4)
Eu1—O1	2.413 (5)

**Fig. 1.**
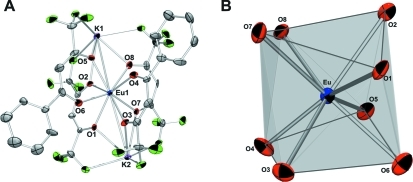
(A) ORTEP diagram of K[Eu(hfac)2(btfac)2] showing the labeling scheme of selected atoms at 30% probability level. Hydrogen atoms are omitted for clarity. (B) Coordination environment around EuIII in K[Eu(hfac)2(btfac)2].

## supplementary crystallographic information

### Comment

Luminescent lanthanide complexes containing β-diketonates have many potential applications (Eliseeva & Bunzli, 2010; de Bettencourt-Dias, 1997; Stanley *et al.*, 2010; Chen & Holliday, 2008). The solid-state crystal structure of K[Eu(hfac)_2_(btfac)_2_], where hfac = 1,1,1,5,5,5-hexafluoro-2,4-pentandionate and btfac = 4,4,4-trifluoro-1-phenyl-1,3-butanedionate, can be seen in Figure 1A. While complexes of the type A[Ln(L)_4_], where A = Na^6+^ or K^+^ and L = acetoacetonate derivatives, have been reported (Nockemann *et al.* (2005); Burns & Danford (1969)), this is the first example of a mixed ligand complex that has been structurally characterized. After removing all atoms not bound to the metal center, it is apparent that the local coordination environment around the trivalent europium ion is a slightly distorted square antiprism (Figure 1B).

### Experimental

The title compound, K[Eu(hfac)_2_(btfac)_2_], was obtained as an unexpected product during the attempted synthesis of a europium tris(β-diketonate) bis(pyrazolyl)pyridine complex in which potassium *tert*-butoxide was used to deprotonate the β-diketone prior to complexation with the lanthanide salt.

### Refinement

The data crystal was twinned, which can result in more than one entry in the reflection file for a given set of h,k,l indices. Twinning can lead to more reflections being used in the refinement than the actual number of unique reflections. The twin law was determined using TwinSolve (Rigaku/MSC, 2002). The twin resulted from a 180 degree rotation about the 0-10 direct lattice direction with twin matrix [-1.000, -0.470, 0.000; -0.001, 1.000, 0.000; 0.000, -0.540, -0.999]. The twin fraction refined to 0.4764 (5).

All H atoms were positioned geometrically and refined using a riding model, with C—H = 0.95 Å and with U_iso_(H) = 1.2 (1.5 for methyl groups) times U_eq_(C).

### Crystal data

**Table:**

K[Eu(C_5_HF_6_O_2_)_2_(C_10_H_6_F_3_O_2_)_2_]	*Z* = 2
*M**_r_* = 1035.47	*F*(000) = 1004
Triclinic, *P*1	*D*_x_ = 1.980 Mg m^−^^3^
Hall symbol: -P 1	Mo *K*α radiation, λ = 0.71069 Å
*a* = 11.737 (2) Å	Cell parameters from 30786 reflections
*b* = 12.468 (2) Å	θ = 4.7–27.5°
*c* = 13.788 (3) Å	µ = 2.07 mm^−^^1^
α = 68.457 (8)°	*T* = 100 K
β = 71.791 (8)°	Block, colourless
γ = 71.726 (8)°	0.12 × 0.06 × 0.03 mm
*V* = 1737.1 (5) Å^3^	

### Data collection

**Table:**

Rigaku AFC12 with Saturn 724+ CCD diffractometer	25336 independent reflections
Radiation source: fine-focus sealed tube	22905 reflections with *I* > 2σ(*I*)
graphite	*R*_int_ = 0.0000
ω–scans	θ_max_ = 25.0°, θ_min_ = 4.7°
Absorption correction: multi-scan (*ABSCOR*; Higashi, 2001)	*h* = −13→13
*T*_min_ = 0.789, *T*_max_ = 0.941	*k* = −14→14
25336 measured reflections	*l* = −16→16

### Refinement

**Table:**

Refinement on *F*^2^	Primary atom site location: structure-invariant direct methods
Least-squares matrix: full	Secondary atom site location: difference Fourier map
*R*[*F*^2^ > 2σ(*F*^2^)] = 0.096	Hydrogen site location: inferred from neighbouring sites
*wR*(*F*^2^) = 0.197	H-atom parameters constrained
*S* = 3.00	*w* = 1/[σ^2^(*F*_o_^2^) + (0.040*P*)^2^] where *P* = (*F*_o_^2^ + 2*F*_c_^2^)/3
25336 reflections	(Δ/σ)_max_ = 0.008
527 parameters	Δρ_max_ = 3.65 e Å^−^^3^
0 restraints	Δρ_min_ = −3.79 e Å^−^^3^

### Special details

**Table:**

Geometry. All esds (except the esd in the dihedral angle between two l.s. planes) are estimated using the full covariance matrix. The cell esds are taken into account individually in the estimation of esds in distances, angles and torsion angles; correlations between esds in cell parameters are only used when they are defined by crystal symmetry. An approximate (isotropic) treatment of cell esds is used for estimating esds involving l.s. planes.
Refinement. Refinement of *F*^2^ against ALL reflections. The weighted *R*-factor wR and goodness of fit *S* are based on *F*^2^, conventional *R*-factors *R* are based on *F*, with *F* set to zero for negative *F*^2^. The threshold expression of *F*^2^ > σ(*F*^2^) is used only for calculating *R*-factors(gt) etc. and is not relevant to the choice of reflections for refinement. *R*-factors based on *F*^2^ are statistically about twice as large as those based on *F*, and *R*- factors based on ALL data will be even larger.

### Fractional atomic coordinates and isotropic or equivalent isotropic displacement parameters (Å^2^)

**Table:**

	*x*	*y*	*z*	*U*_iso_*/*U*_eq_	
C1	0.1917 (8)	0.5647 (8)	−0.0670 (7)	0.046 (2)	
C2	0.1678 (6)	0.5603 (6)	0.0491 (5)	0.0270 (16)	
C3	0.0581 (7)	0.6211 (6)	0.0938 (6)	0.0309 (17)	
H3	0.0018	0.6697	0.0497	0.037*	
C4	0.0261 (6)	0.6153 (6)	0.1978 (6)	0.0258 (16)	
C5	−0.0953 (7)	0.6967 (7)	0.2354 (6)	0.0380 (19)	
C6	0.6359 (8)	0.1771 (7)	0.1235 (6)	0.041 (2)	
C7	0.5469 (7)	0.2239 (6)	0.2119 (6)	0.0338 (17)	
C8	0.5666 (7)	0.1706 (7)	0.3098 (7)	0.039 (2)	
H8	0.6384	0.1092	0.3191	0.047*	
C9	0.4860 (6)	0.2006 (6)	0.4008 (6)	0.0296 (17)	
C10	0.5163 (8)	0.1435 (6)	0.5104 (6)	0.0381 (19)	
C11	0.4273 (8)	0.1526 (9)	0.5946 (6)	0.066 (3)	
H25	0.3452	0.1863	0.5858	0.079*	
C12	0.4490 (10)	0.1155 (9)	0.6927 (8)	0.065 (3)	
H26	0.3856	0.1315	0.7517	0.078*	
C13	0.5696 (10)	0.0513 (9)	0.7066 (8)	0.058 (3)	
H27	0.5886	0.0198	0.7752	0.070*	
C14	0.6554 (8)	0.0375 (9)	0.6180 (8)	0.057 (3)	
H28	0.7372	−0.0031	0.6249	0.069*	
C15	0.6304 (9)	0.0789 (9)	0.5191 (7)	0.063 (3)	
H29	0.6909	0.0628	0.4587	0.076*	
C16	0.0084 (8)	0.1836 (7)	0.5500 (6)	0.041 (2)	
C17	0.0708 (7)	0.2337 (6)	0.4328 (6)	0.0319 (18)	
C18	0.0681 (9)	0.1848 (8)	0.3593 (7)	0.050 (3)	
H15	0.0174	0.1301	0.3809	0.059*	
C19	0.1342 (8)	0.2112 (6)	0.2585 (7)	0.040 (2)	
C20	0.1338 (7)	0.1522 (6)	0.1815 (6)	0.0357 (18)	
C21	0.2254 (11)	0.1550 (9)	0.0945 (7)	0.079 (4)	
H16	0.2851	0.1988	0.0802	0.095*	
C22	0.2341 (10)	0.0944 (8)	0.0250 (8)	0.066 (3)	
H17	0.3015	0.0934	−0.0349	0.079*	
C23	0.1444 (12)	0.0361 (9)	0.0438 (9)	0.076 (4)	
H18	0.1508	−0.0062	−0.0034	0.091*	
C24	0.0410 (10)	0.0374 (8)	0.1331 (8)	0.068 (3)	
H19	−0.0242	0.0011	0.1450	0.081*	
C25	0.0430 (9)	0.0959 (8)	0.2015 (7)	0.049 (2)	
H20	−0.0211	0.0963	0.2639	0.059*	
C26	0.2817 (8)	0.5623 (8)	0.5313 (7)	0.045 (2)	
C27	0.3058 (7)	0.5583 (6)	0.4158 (6)	0.0352 (18)	
C28	0.3889 (6)	0.6178 (6)	0.3388 (6)	0.0266 (16)	
H22	0.4258	0.6631	0.3574	0.032*	
C29	0.4211 (6)	0.6142 (6)	0.2347 (6)	0.0283 (16)	
C30	0.5014 (7)	0.6967 (7)	0.1520 (6)	0.0388 (19)	
Eu1	0.27159 (3)	0.40232 (3)	0.27527 (3)	0.02047 (10)	
F1	0.3069 (4)	0.5677 (4)	−0.1188 (3)	0.0435 (11)	
F2	0.1728 (5)	0.4659 (5)	−0.0700 (4)	0.0657 (17)	
F3	0.1194 (5)	0.6532 (6)	−0.1202 (4)	0.088 (2)	
F4	−0.1848 (4)	0.6928 (5)	0.2024 (4)	0.0598 (14)	
F5	−0.0824 (5)	0.8077 (4)	0.2013 (4)	0.0570 (15)	
F6	−0.1285 (4)	0.6694 (4)	0.3418 (3)	0.0407 (11)	
F7	0.6624 (4)	0.2662 (3)	0.0350 (3)	0.0412 (11)	
F8	0.7376 (5)	0.1090 (5)	0.1463 (4)	0.0737 (17)	
F9	0.5835 (5)	0.1168 (4)	0.0933 (4)	0.0519 (13)	
F10	0.0912 (5)	0.1121 (3)	0.6036 (4)	0.0563 (14)	
F11	−0.0526 (4)	0.2664 (3)	0.5961 (3)	0.0385 (10)	
F12	−0.0690 (5)	0.1203 (4)	0.5625 (4)	0.0638 (15)	
F13	0.5488 (4)	0.6708 (4)	0.0626 (3)	0.0395 (10)	
F14	0.4382 (5)	0.8067 (4)	0.1321 (4)	0.0584 (16)	
F15	0.5956 (5)	0.6911 (5)	0.1884 (4)	0.0709 (16)	
F16	0.1628 (4)	0.5698 (4)	0.5792 (3)	0.0427 (11)	
F17	0.3433 (5)	0.4672 (5)	0.5884 (4)	0.0657 (17)	
F18	0.3105 (6)	0.6547 (5)	0.5334 (4)	0.0800 (19)	
O1	0.2551 (4)	0.4986 (5)	0.0915 (4)	0.0251 (12)	
O2	0.0837 (4)	0.5516 (4)	0.2704 (4)	0.0269 (11)	
O3	0.4609 (4)	0.3098 (4)	0.1823 (4)	0.0312 (11)	
O4	0.3868 (4)	0.2735 (4)	0.3960 (4)	0.0358 (12)	
O5	0.1244 (4)	0.3130 (4)	0.4191 (3)	0.0291 (11)	
O6	0.2076 (5)	0.2811 (4)	0.2196 (4)	0.0368 (12)	
O7	0.3927 (4)	0.5503 (4)	0.1993 (4)	0.0273 (11)	
O8	0.2463 (4)	0.4963 (5)	0.4063 (4)	0.0302 (13)	
K1	0.0000	0.5000	0.5000	0.0293 (6)	
K2	0.5000	0.5000	0.0000	0.0270 (6)	

### Atomic displacement parameters (Å^2^)

**Table:**

	*U*^11^	*U*^22^	*U*^33^	*U*^12^	*U*^13^	*U*^23^
C1	0.042 (5)	0.060 (6)	0.039 (5)	0.001 (4)	−0.019 (4)	−0.020 (4)
C2	0.023 (4)	0.037 (4)	0.021 (4)	−0.013 (3)	−0.003 (3)	−0.007 (3)
C3	0.031 (4)	0.033 (4)	0.022 (4)	−0.006 (3)	−0.003 (3)	−0.003 (3)
C4	0.018 (4)	0.018 (3)	0.035 (4)	0.003 (3)	−0.005 (3)	−0.008 (3)
C5	0.037 (5)	0.050 (5)	0.027 (4)	−0.004 (4)	−0.013 (4)	−0.012 (4)
C6	0.044 (5)	0.040 (5)	0.029 (4)	0.002 (4)	0.001 (4)	−0.014 (4)
C7	0.029 (4)	0.028 (4)	0.041 (5)	−0.002 (3)	−0.002 (4)	−0.015 (3)
C8	0.026 (4)	0.030 (4)	0.049 (5)	0.005 (3)	0.000 (4)	−0.013 (4)
C9	0.026 (4)	0.033 (4)	0.025 (4)	−0.009 (3)	0.002 (3)	−0.007 (3)
C10	0.051 (5)	0.023 (4)	0.034 (4)	−0.004 (3)	−0.010 (4)	−0.003 (3)
C11	0.037 (5)	0.099 (8)	0.025 (5)	0.013 (5)	−0.010 (4)	0.001 (5)
C12	0.064 (7)	0.074 (7)	0.051 (6)	0.007 (5)	−0.020 (5)	−0.026 (5)
C13	0.060 (7)	0.075 (7)	0.053 (6)	−0.007 (5)	−0.030 (6)	−0.026 (5)
C14	0.030 (5)	0.076 (7)	0.058 (6)	−0.015 (4)	−0.018 (5)	−0.001 (5)
C15	0.046 (6)	0.071 (7)	0.024 (5)	0.032 (5)	−0.008 (4)	0.001 (4)
C16	0.057 (5)	0.027 (4)	0.032 (4)	−0.020 (4)	0.003 (4)	−0.001 (3)
C17	0.034 (4)	0.017 (4)	0.033 (4)	−0.009 (3)	0.011 (3)	−0.007 (3)
C18	0.064 (6)	0.047 (6)	0.049 (6)	−0.033 (5)	0.014 (5)	−0.031 (4)
C19	0.058 (5)	0.032 (4)	0.039 (5)	−0.023 (4)	−0.015 (4)	−0.009 (4)
C20	0.048 (5)	0.034 (4)	0.029 (4)	−0.025 (4)	0.001 (4)	−0.007 (3)
C21	0.141 (10)	0.098 (8)	0.032 (5)	−0.096 (8)	0.022 (6)	−0.034 (5)
C22	0.080 (7)	0.041 (5)	0.072 (7)	−0.021 (5)	0.010 (6)	−0.028 (5)
C23	0.104 (9)	0.052 (6)	0.068 (7)	−0.019 (6)	0.005 (7)	−0.032 (6)
C24	0.088 (8)	0.052 (6)	0.059 (7)	−0.035 (5)	0.002 (6)	−0.010 (5)
C25	0.070 (6)	0.060 (6)	0.033 (5)	−0.044 (5)	0.001 (4)	−0.018 (4)
C26	0.050 (6)	0.058 (6)	0.040 (5)	−0.017 (5)	−0.011 (5)	−0.023 (4)
C27	0.030 (4)	0.030 (4)	0.055 (5)	0.004 (3)	−0.018 (4)	−0.025 (4)
C28	0.017 (4)	0.028 (4)	0.045 (5)	−0.011 (3)	−0.014 (3)	−0.012 (3)
C29	0.026 (4)	0.031 (4)	0.031 (4)	−0.003 (3)	−0.014 (3)	−0.009 (3)
C30	0.041 (5)	0.047 (5)	0.040 (5)	−0.029 (4)	−0.008 (4)	−0.009 (4)
Eu1	0.01748 (16)	0.02274 (16)	0.02001 (16)	−0.00665 (12)	0.00050 (11)	−0.00718 (13)
F1	0.047 (3)	0.057 (3)	0.027 (2)	−0.017 (2)	−0.004 (2)	−0.013 (2)
F2	0.067 (4)	0.100 (6)	0.058 (4)	−0.045 (3)	0.001 (3)	−0.046 (3)
F3	0.066 (4)	0.129 (5)	0.034 (3)	0.036 (4)	−0.023 (3)	−0.024 (3)
F4	0.028 (3)	0.092 (4)	0.074 (4)	0.013 (2)	−0.025 (3)	−0.052 (3)
F5	0.077 (4)	0.025 (3)	0.053 (3)	−0.002 (2)	−0.005 (3)	−0.008 (2)
F6	0.027 (2)	0.041 (2)	0.044 (3)	0.0052 (18)	−0.001 (2)	−0.018 (2)
F7	0.037 (3)	0.036 (2)	0.039 (3)	−0.0106 (19)	0.012 (2)	−0.014 (2)
F8	0.054 (3)	0.080 (4)	0.051 (3)	0.039 (3)	−0.011 (3)	−0.025 (3)
F9	0.069 (3)	0.037 (2)	0.045 (3)	−0.013 (2)	0.011 (3)	−0.027 (2)
F10	0.068 (3)	0.036 (2)	0.038 (3)	0.000 (3)	0.001 (3)	−0.001 (2)
F11	0.042 (3)	0.042 (2)	0.030 (2)	−0.017 (2)	0.006 (2)	−0.016 (2)
F12	0.096 (4)	0.060 (3)	0.043 (3)	−0.060 (3)	0.019 (3)	−0.018 (2)
F13	0.037 (2)	0.047 (3)	0.033 (2)	−0.022 (2)	0.007 (2)	−0.011 (2)
F14	0.092 (4)	0.031 (3)	0.043 (3)	−0.021 (3)	0.003 (3)	−0.011 (2)
F15	0.067 (4)	0.109 (4)	0.053 (3)	−0.066 (3)	−0.008 (3)	−0.008 (3)
F16	0.038 (3)	0.057 (3)	0.031 (2)	−0.011 (2)	0.006 (2)	−0.023 (2)
F17	0.051 (3)	0.104 (6)	0.037 (3)	0.000 (3)	−0.024 (3)	−0.017 (3)
F18	0.120 (5)	0.106 (5)	0.051 (3)	−0.075 (4)	0.008 (3)	−0.046 (3)
O1	0.024 (3)	0.029 (2)	0.031 (3)	−0.015 (2)	−0.001 (3)	−0.015 (2)
O2	0.029 (3)	0.029 (3)	0.023 (3)	−0.013 (2)	0.003 (2)	−0.009 (2)
O3	0.023 (3)	0.028 (3)	0.035 (3)	−0.003 (2)	0.004 (2)	−0.011 (2)
O4	0.028 (3)	0.038 (3)	0.030 (3)	0.007 (2)	−0.004 (2)	−0.012 (2)
O5	0.038 (3)	0.033 (3)	0.018 (2)	−0.014 (2)	0.001 (2)	−0.011 (2)
O6	0.041 (3)	0.044 (3)	0.034 (3)	−0.020 (2)	0.002 (2)	−0.020 (2)
O7	0.022 (3)	0.034 (3)	0.032 (3)	−0.012 (2)	−0.003 (2)	−0.015 (2)
O8	0.028 (3)	0.027 (2)	0.041 (4)	−0.010 (2)	−0.004 (3)	−0.016 (2)
K1	0.0263 (13)	0.0289 (11)	0.0318 (18)	−0.0071 (10)	−0.0013 (13)	−0.0118 (9)
K2	0.0237 (12)	0.0352 (11)	0.0197 (16)	−0.0096 (10)	0.0031 (12)	−0.0095 (9)

### Geometric parameters (Å, °)

**Table:**

C1—F3	1.297 (9)	C17—O5	1.260 (8)
C1—F1	1.322 (9)	C17—C18	1.373 (11)
C1—F2	1.335 (10)	C18—C19	1.340 (11)
C1—C2	1.519 (10)	C18—H15	0.9500
C2—O1	1.239 (8)	C19—O6	1.276 (9)
C2—C3	1.359 (10)	C19—C20	1.499 (10)
C3—C4	1.345 (10)	C20—C21	1.335 (11)
C3—H3	0.9500	C20—C25	1.360 (11)
C4—O2	1.261 (8)	C21—C22	1.386 (12)
C4—C5	1.531 (10)	C21—H16	0.9500
C5—F4	1.288 (8)	C22—C23	1.369 (15)
C5—F5	1.330 (9)	C22—H17	0.9500
C5—F6	1.334 (8)	C23—C24	1.435 (14)
C6—F8	1.284 (9)	C23—H18	0.9500
C6—F9	1.339 (10)	C24—C25	1.396 (12)
C6—F7	1.343 (8)	C24—H19	0.9500
C6—C7	1.504 (10)	C25—H20	0.9500
C7—O3	1.262 (9)	C26—F17	1.296 (10)
C7—C8	1.325 (11)	C26—F18	1.310 (9)
C8—C9	1.410 (10)	C26—F16	1.334 (9)
C8—H8	0.9500	C26—C27	1.545 (11)
C9—O4	1.236 (8)	C27—O8	1.253 (8)
C9—C10	1.518 (10)	C27—C28	1.363 (10)
C10—C11	1.311 (11)	C28—C29	1.380 (10)
C10—C15	1.345 (12)	C28—H22	0.9500
C11—C12	1.338 (12)	C29—O7	1.239 (8)
C11—H25	0.9500	C29—C30	1.524 (10)
C12—C13	1.423 (14)	C30—F13	1.297 (8)
C12—H26	0.9500	C30—F14	1.312 (9)
C13—C14	1.347 (13)	C30—F15	1.323 (9)
C13—H27	0.9500	Eu1—O4	2.294 (5)
C14—C15	1.361 (12)	Eu1—O6	2.315 (5)
C14—H28	0.9500	Eu1—O5	2.367 (4)
C15—H29	0.9500	Eu1—O3	2.368 (4)
C16—F10	1.307 (10)	Eu1—O8	2.394 (5)
C16—F11	1.310 (8)	Eu1—O2	2.400 (5)
C16—F12	1.315 (9)	Eu1—O7	2.412 (4)
C16—C17	1.531 (10)	Eu1—O1	2.413 (5)
			
F3—C1—F1	108.5 (7)	C16—F11—K1	119.4 (4)
F3—C1—F2	107.2 (7)	C30—F13—K2	127.8 (4)
F1—C1—F2	106.0 (6)	C26—F16—K1	121.0 (4)
F3—C1—C2	113.2 (7)	C2—O1—Eu1	133.2 (4)
F1—C1—C2	112.7 (7)	C2—O1—K2	125.6 (4)
F2—C1—C2	108.8 (7)	Eu1—O1—K2	98.37 (15)
O1—C2—C3	128.0 (7)	C4—O2—Eu1	133.7 (4)
O1—C2—C1	113.1 (6)	C4—O2—K1	129.6 (4)
C3—C2—C1	118.9 (7)	Eu1—O2—K1	95.34 (15)
C4—C3—C2	122.7 (7)	C7—O3—Eu1	133.5 (5)
C4—C3—H3	118.7	C7—O3—K2	122.7 (4)
C2—C3—H3	118.7	Eu1—O3—K2	98.83 (15)
O2—C4—C3	128.6 (6)	C9—O4—Eu1	141.3 (5)
O2—C4—C5	114.3 (6)	C17—O5—Eu1	132.8 (4)
C3—C4—C5	117.1 (6)	C17—O5—K1	120.0 (4)
F4—C5—F5	109.1 (6)	Eu1—O5—K1	99.91 (15)
F4—C5—F6	107.6 (6)	C19—O6—Eu1	138.0 (5)
F5—C5—F6	105.5 (6)	C29—O7—Eu1	134.9 (5)
F4—C5—C4	112.5 (6)	C29—O7—K2	128.1 (4)
F5—C5—C4	110.0 (6)	Eu1—O7—K2	95.88 (14)
F6—C5—C4	111.9 (6)	C27—O8—Eu1	133.6 (5)
F8—C6—F9	107.5 (7)	C27—O8—K1	124.7 (4)
F8—C6—F7	108.2 (7)	Eu1—O8—K1	98.81 (14)
F9—C6—F7	104.3 (6)	O5^i^—K1—O5	180.0
F8—C6—C7	115.2 (7)	O5^i^—K1—O8^i^	65.46 (14)
F9—C6—C7	110.2 (7)	O5—K1—O8^i^	114.54 (14)
F7—C6—C7	110.9 (6)	O5^i^—K1—O8	114.54 (14)
O3—C7—C8	128.2 (7)	O5—K1—O8	65.46 (14)
O3—C7—C6	114.5 (7)	O8^i^—K1—O8	180.0
C8—C7—C6	117.4 (7)	O5^i^—K1—F6	74.59 (13)
C7—C8—C9	123.1 (7)	O5—K1—F6	105.41 (13)
C7—C8—H8	118.4	O8^i^—K1—F6	75.14 (14)
C9—C8—H8	118.4	O8—K1—F6	104.86 (14)
O4—C9—C8	122.1 (7)	O5^i^—K1—F6^i^	105.41 (13)
O4—C9—C10	116.4 (6)	O5—K1—F6^i^	74.59 (13)
C8—C9—C10	121.5 (7)	O8^i^—K1—F6^i^	104.86 (14)
C11—C10—C15	121.9 (8)	O8—K1—F6^i^	75.14 (14)
C11—C10—C9	118.3 (7)	F6—K1—F6^i^	180.0
C15—C10—C9	119.6 (7)	O5^i^—K1—O2^i^	62.26 (13)
C10—C11—C12	121.7 (9)	O5—K1—O2^i^	117.74 (13)
C10—C11—H25	119.1	O8^i^—K1—O2^i^	60.32 (14)
C12—C11—H25	119.1	O8—K1—O2^i^	119.68 (14)
C11—C12—C13	118.4 (9)	F6—K1—O2^i^	127.09 (12)
C11—C12—H26	120.8	F6^i^—K1—O2^i^	52.91 (12)
C13—C12—H26	120.8	O5^i^—K1—O2	117.74 (13)
C14—C13—C12	116.9 (9)	O5—K1—O2	62.26 (13)
C14—C13—H27	121.5	O8^i^—K1—O2	119.68 (14)
C12—C13—H27	121.5	O8—K1—O2	60.32 (14)
C13—C14—C15	123.0 (9)	F6—K1—O2	52.91 (12)
C13—C14—H28	118.5	F6^i^—K1—O2	127.09 (12)
C15—C14—H28	118.5	O2^i^—K1—O2	180.000 (1)
C10—C15—C14	117.2 (8)	O5^i^—K1—F11^i^	56.45 (12)
C10—C15—H29	121.4	O5—K1—F11^i^	123.55 (12)
C14—C15—H29	121.4	O8^i^—K1—F11^i^	113.43 (13)
F10—C16—F11	108.0 (7)	O8—K1—F11^i^	66.57 (13)
F10—C16—F12	105.7 (6)	F6—K1—F11^i^	61.25 (13)
F11—C16—F12	107.2 (6)	F6^i^—K1—F11^i^	118.75 (13)
F10—C16—C17	109.8 (7)	O2^i^—K1—F11^i^	110.84 (11)
F11—C16—C17	112.5 (6)	O2—K1—F11^i^	69.16 (11)
F12—C16—C17	113.2 (7)	O5^i^—K1—F11	123.55 (12)
O5—C17—C18	128.8 (7)	O5—K1—F11	56.45 (12)
O5—C17—C16	112.7 (7)	O8^i^—K1—F11	66.57 (13)
C18—C17—C16	118.4 (7)	O8—K1—F11	113.43 (13)
C19—C18—C17	122.5 (8)	F6—K1—F11	118.75 (13)
C19—C18—H15	118.7	F6^i^—K1—F11	61.25 (13)
C17—C18—H15	118.7	O2^i^—K1—F11	69.16 (11)
O6—C19—C18	125.3 (7)	O2—K1—F11	110.84 (11)
O6—C19—C20	113.3 (7)	F11^i^—K1—F11	180.000 (1)
C18—C19—C20	121.3 (7)	O5^i^—K1—F16^i^	111.12 (13)
C21—C20—C25	121.3 (7)	O5—K1—F16^i^	68.88 (13)
C21—C20—C19	118.3 (7)	O8^i^—K1—F16^i^	55.40 (12)
C25—C20—C19	120.3 (7)	O8—K1—F16^i^	124.60 (12)
C20—C21—C22	120.5 (8)	F6—K1—F16^i^	59.70 (13)
C20—C21—H16	119.7	F6^i^—K1—F16^i^	120.30 (13)
C22—C21—H16	119.7	O2^i^—K1—F16^i^	108.34 (12)
C23—C22—C21	119.3 (9)	O2—K1—F16^i^	71.66 (12)
C23—C22—H17	120.4	F11^i^—K1—F16^i^	120.65 (12)
C21—C22—H17	120.4	F11—K1—F16^i^	59.35 (12)
C22—C23—C24	121.4 (10)	O5^i^—K1—F16	68.88 (13)
C22—C23—H18	119.3	O5—K1—F16	111.12 (13)
C24—C23—H18	119.3	O8^i^—K1—F16	124.60 (12)
C25—C24—C23	115.3 (10)	O8—K1—F16	55.40 (12)
C25—C24—H19	122.4	F6—K1—F16	120.30 (13)
C23—C24—H19	122.4	F6^i^—K1—F16	59.70 (13)
C20—C25—C24	122.0 (8)	O2^i^—K1—F16	71.66 (12)
C20—C25—H20	119.0	O2—K1—F16	108.34 (12)
C24—C25—H20	119.0	F11^i^—K1—F16	59.35 (12)
F17—C26—F18	109.0 (7)	F11—K1—F16	120.65 (12)
F17—C26—F16	107.3 (7)	F16^i^—K1—F16	180.00 (14)
F18—C26—F16	106.6 (7)	O1^ii^—K2—O1	180.0
F17—C26—C27	111.0 (7)	O1^ii^—K2—O3^ii^	65.99 (14)
F18—C26—C27	111.8 (7)	O1—K2—O3^ii^	114.01 (14)
F16—C26—C27	110.9 (7)	O1^ii^—K2—O3	114.01 (14)
O8—C27—C28	128.3 (7)	O1—K2—O3	65.99 (14)
O8—C27—C26	113.8 (7)	O3^ii^—K2—O3	180.00 (16)
C28—C27—C26	117.9 (7)	O1^ii^—K2—F13^ii^	105.49 (12)
C27—C28—C29	122.0 (6)	O1—K2—F13^ii^	74.51 (12)
C27—C28—H22	119.0	O3^ii^—K2—F13^ii^	107.25 (13)
C29—C28—H22	119.0	O3—K2—F13^ii^	72.75 (13)
O7—C29—C28	127.5 (7)	O1^ii^—K2—F13	74.51 (12)
O7—C29—C30	114.9 (6)	O1—K2—F13	105.49 (12)
C28—C29—C30	117.6 (6)	O3^ii^—K2—F13	72.75 (13)
F13—C30—F14	109.0 (6)	O3—K2—F13	107.25 (13)
F13—C30—F15	106.0 (6)	F13^ii^—K2—F13	180.0
F14—C30—F15	107.1 (6)	O1^ii^—K2—O7^ii^	60.91 (12)
F13—C30—C29	113.6 (6)	O1—K2—O7^ii^	119.09 (12)
F14—C30—C29	110.4 (6)	O3^ii^—K2—O7^ii^	62.65 (13)
F15—C30—C29	110.5 (6)	O3—K2—O7^ii^	117.35 (13)
O4—Eu1—O6	104.33 (17)	F13^ii^—K2—O7^ii^	53.95 (11)
O4—Eu1—O5	76.78 (17)	F13—K2—O7^ii^	126.05 (11)
O6—Eu1—O5	71.91 (16)	O1^ii^—K2—O7	119.09 (12)
O4—Eu1—O3	70.88 (16)	O1—K2—O7	60.91 (12)
O6—Eu1—O3	78.60 (17)	O3^ii^—K2—O7	117.35 (13)
O5—Eu1—O3	128.55 (14)	O3—K2—O7	62.65 (13)
O4—Eu1—O8	72.72 (18)	F13^ii^—K2—O7	126.05 (11)
O6—Eu1—O8	149.11 (16)	F13—K2—O7	53.95 (11)
O5—Eu1—O8	77.60 (16)	O7^ii^—K2—O7	180.0
O3—Eu1—O8	126.22 (16)	O1^ii^—K2—F7	66.14 (13)
O4—Eu1—O2	139.43 (16)	O1—K2—F7	113.86 (13)
O6—Eu1—O2	94.56 (17)	O3^ii^—K2—F7	124.48 (12)
O5—Eu1—O2	75.54 (15)	O3—K2—F7	55.52 (12)
O3—Eu1—O2	149.11 (16)	F13^ii^—K2—F7	61.71 (13)
O8—Eu1—O2	72.79 (16)	F13—K2—F7	118.29 (13)
O4—Eu1—O7	97.01 (17)	O7^ii^—K2—F7	70.51 (12)
O6—Eu1—O7	138.70 (16)	O7—K2—F7	109.49 (12)
O5—Eu1—O7	148.45 (15)	O1^ii^—K2—F7^ii^	113.86 (13)
O3—Eu1—O7	75.60 (15)	O1—K2—F7^ii^	66.14 (13)
O8—Eu1—O7	71.08 (16)	O3^ii^—K2—F7^ii^	55.52 (12)
O2—Eu1—O7	91.44 (13)	O3—K2—F7^ii^	124.48 (12)
O4—Eu1—O1	149.01 (16)	F13^ii^—K2—F7^ii^	118.29 (13)
O6—Eu1—O1	71.28 (16)	F13—K2—F7^ii^	61.71 (13)
O5—Eu1—O1	127.16 (15)	O7^ii^—K2—F7^ii^	109.49 (12)
O3—Eu1—O1	78.24 (17)	O7—K2—F7^ii^	70.51 (12)
O8—Eu1—O1	126.86 (13)	F7—K2—F7^ii^	180.0
O2—Eu1—O1	71.08 (16)	O1^ii^—K2—F1	125.14 (13)
O7—Eu1—O1	72.26 (15)	O1—K2—F1	54.86 (13)
O4—Eu1—K2	107.55 (12)	O3^ii^—K2—F1	69.57 (13)
O6—Eu1—K2	93.08 (12)	O3—K2—F1	110.43 (13)
O5—Eu1—K2	164.99 (11)	F13^ii^—K2—F1	59.77 (12)
O3—Eu1—K2	44.52 (11)	F13—K2—F1	120.23 (12)
O8—Eu1—K2	117.38 (12)	O7^ii^—K2—F1	71.04 (12)
O2—Eu1—K2	106.88 (10)	O7—K2—F1	108.96 (12)
O7—Eu1—K2	46.38 (11)	F7—K2—F1	121.13 (12)
O1—Eu1—K2	44.11 (10)	F7^ii^—K2—F1	58.87 (12)
O4—Eu1—K1	92.65 (12)	O1^ii^—K2—F1^ii^	54.86 (13)
O6—Eu1—K1	106.80 (12)	O1—K2—F1^ii^	125.14 (13)
O5—Eu1—K1	43.66 (10)	O3^ii^—K2—F1^ii^	110.43 (13)
O3—Eu1—K1	163.53 (12)	O3—K2—F1^ii^	69.57 (13)
O8—Eu1—K1	44.13 (11)	F13^ii^—K2—F1^ii^	120.23 (12)
O2—Eu1—K1	47.16 (11)	F13—K2—F1^ii^	59.77 (12)
O7—Eu1—K1	107.07 (11)	O7^ii^—K2—F1^ii^	108.96 (12)
O1—Eu1—K1	118.19 (12)	O7—K2—F1^ii^	71.04 (12)
K2—Eu1—K1	147.309 (12)	F7—K2—F1^ii^	58.87 (12)
C1—F1—K2	120.3 (4)	F7^ii^—K2—F1^ii^	121.13 (12)
C5—F6—K1	129.9 (4)	F1—K2—F1^ii^	180.0
C6—F7—K2	121.4 (4)		
			
F3—C1—C2—O1	−161.2 (7)	O7—C29—C30—F15	−132.4 (7)
F1—C1—C2—O1	−37.6 (9)	C28—C29—C30—F15	47.2 (9)
F2—C1—C2—O1	79.7 (8)	C3—C2—O1—Eu1	20.3 (11)
F3—C1—C2—C3	19.0 (11)	C1—C2—O1—Eu1	−159.5 (5)
F1—C1—C2—C3	142.6 (7)	O4—Eu1—O1—C2	170.4 (6)
F2—C1—C2—C3	−100.1 (8)	O6—Eu1—O1—C2	83.7 (6)
O1—C2—C3—C4	−4.5 (12)	O5—Eu1—O1—C2	35.6 (7)
C1—C2—C3—C4	175.2 (7)	O3—Eu1—O1—C2	165.5 (6)
C2—C3—C4—O2	−4.5 (12)	O8—Eu1—O1—C2	−67.8 (6)
C2—C3—C4—C5	174.8 (7)	O2—Eu1—O1—C2	−18.2 (6)
O2—C4—C5—F4	−132.8 (7)	O7—Eu1—O1—C2	−116.1 (6)
C3—C4—C5—F4	47.8 (9)	C3—C4—O2—Eu1	−2.8 (11)
O2—C4—C5—F5	105.4 (7)	C5—C4—O2—Eu1	177.9 (4)
C3—C4—C5—F5	−74.0 (9)	O4—Eu1—O2—C4	−176.9 (5)
O2—C4—C5—F6	−11.5 (9)	O6—Eu1—O2—C4	−58.5 (6)
C3—C4—C5—F6	169.1 (6)	O5—Eu1—O2—C4	−128.5 (6)
F8—C6—C7—O3	165.1 (7)	O3—Eu1—O2—C4	16.8 (7)
F9—C6—C7—O3	−73.2 (8)	O8—Eu1—O2—C4	150.2 (6)
F7—C6—C7—O3	41.8 (10)	O7—Eu1—O2—C4	80.6 (6)
F8—C6—C7—C8	−15.8 (11)	O1—Eu1—O2—C4	9.9 (6)
F9—C6—C7—C8	106.0 (9)	C8—C7—O3—Eu1	−13.1 (12)
F7—C6—C7—C8	−139.1 (8)	C6—C7—O3—Eu1	165.9 (5)
O3—C7—C8—C9	3.2 (14)	O4—Eu1—O3—C7	10.3 (6)
C6—C7—C8—C9	−175.8 (7)	O6—Eu1—O3—C7	−99.4 (6)
C7—C8—C9—O4	5.1 (13)	O5—Eu1—O3—C7	−43.9 (7)
C7—C8—C9—C10	−175.6 (8)	O8—Eu1—O3—C7	60.2 (7)
O4—C9—C10—C11	15.6 (11)	O2—Eu1—O3—C7	−179.1 (6)
C8—C9—C10—C11	−163.8 (9)	O7—Eu1—O3—C7	113.1 (7)
O4—C9—C10—C15	−168.9 (8)	O1—Eu1—O3—C7	−172.4 (6)
C8—C9—C10—C15	11.7 (12)	C8—C9—O4—Eu1	−3.8 (12)
C15—C10—C11—C12	11.7 (16)	C10—C9—O4—Eu1	176.9 (5)
C9—C10—C11—C12	−172.9 (9)	O6—Eu1—O4—C9	70.1 (8)
C10—C11—C12—C13	−8.0 (17)	O5—Eu1—O4—C9	137.2 (8)
C11—C12—C13—C14	3.1 (16)	O3—Eu1—O4—C9	−2.2 (7)
C12—C13—C14—C15	−1.8 (16)	O8—Eu1—O4—C9	−141.9 (8)
C11—C10—C15—C14	−9.7 (16)	O2—Eu1—O4—C9	−174.8 (7)
C9—C10—C15—C14	175.0 (8)	O7—Eu1—O4—C9	−74.2 (8)
C13—C14—C15—C10	4.9 (16)	O1—Eu1—O4—C9	−7.3 (10)
F10—C16—C17—O5	−75.9 (8)	C18—C17—O5—Eu1	−10.9 (13)
F11—C16—C17—O5	44.5 (9)	C16—C17—O5—Eu1	165.1 (5)
F12—C16—C17—O5	166.3 (7)	O4—Eu1—O5—C17	−103.0 (7)
F10—C16—C17—C18	100.6 (9)	O6—Eu1—O5—C17	7.1 (6)
F11—C16—C17—C18	−139.0 (8)	O3—Eu1—O5—C17	−51.1 (7)
F12—C16—C17—C18	−17.2 (11)	O8—Eu1—O5—C17	−177.9 (7)
O5—C17—C18—C19	5.4 (15)	O2—Eu1—O5—C17	107.0 (7)
C16—C17—C18—C19	−170.5 (8)	O7—Eu1—O5—C17	175.2 (6)
C17—C18—C19—O6	1.4 (15)	O1—Eu1—O5—C17	55.0 (7)
C17—C18—C19—C20	177.2 (8)	C18—C19—O6—Eu1	−2.4 (14)
O6—C19—C20—C21	15.3 (12)	C20—C19—O6—Eu1	−178.4 (5)
C18—C19—C20—C21	−161.0 (10)	O4—Eu1—O6—C19	69.7 (8)
O6—C19—C20—C25	−165.8 (8)	O5—Eu1—O6—C19	−0.9 (7)
C18—C19—C20—C25	18.0 (13)	O3—Eu1—O6—C19	136.4 (8)
C25—C20—C21—C22	−3.7 (17)	O8—Eu1—O6—C19	−10.5 (10)
C19—C20—C21—C22	175.3 (9)	O2—Eu1—O6—C19	−74.1 (8)
C20—C21—C22—C23	3.0 (18)	O7—Eu1—O6—C19	−171.5 (7)
C21—C22—C23—C24	0.7 (18)	O1—Eu1—O6—C19	−142.3 (8)
C22—C23—C24—C25	−3.6 (18)	C28—C29—O7—Eu1	0.9 (11)
C21—C20—C25—C24	0.5 (15)	C30—C29—O7—Eu1	−179.5 (5)
C19—C20—C25—C24	−178.4 (9)	O4—Eu1—O7—C29	−60.7 (6)
C23—C24—C25—C20	3.0 (16)	O6—Eu1—O7—C29	178.1 (6)
F17—C26—C27—O8	79.2 (8)	O5—Eu1—O7—C29	15.4 (8)
F18—C26—C27—O8	−158.8 (7)	O3—Eu1—O7—C29	−128.9 (6)
F16—C26—C27—O8	−40.0 (9)	O8—Eu1—O7—C29	8.3 (6)
F17—C26—C27—C28	−99.1 (8)	O2—Eu1—O7—C29	79.5 (6)
F18—C26—C27—C28	22.8 (10)	O1—Eu1—O7—C29	149.1 (6)
F16—C26—C27—C28	141.7 (7)	C28—C27—O8—Eu1	18.7 (12)
O8—C27—C28—C29	−1.5 (12)	C26—C27—O8—Eu1	−159.4 (5)
C26—C27—C28—C29	176.6 (6)	O4—Eu1—O8—C27	86.5 (7)
C27—C28—C29—O7	−8.5 (12)	O6—Eu1—O8—C27	175.6 (6)
C27—C28—C29—C30	172.0 (7)	O5—Eu1—O8—C27	166.3 (7)
O7—C29—C30—F13	−13.5 (10)	O3—Eu1—O8—C27	37.2 (7)
C28—C29—C30—F13	166.1 (6)	O2—Eu1—O8—C27	−115.2 (7)
O7—C29—C30—F14	109.3 (7)	O7—Eu1—O8—C27	−17.5 (6)
C28—C29—C30—F14	−71.1 (9)	O1—Eu1—O8—C27	−66.3 (7)

Symmetry codes: (i) −*x*, −*y*+1, −*z*+1; (ii) −*x*+1, −*y*+1, −*z*.
